# Data on skeletal muscle apoptosis, autophagy, and morphology in mice treated with doxorubicin

**DOI:** 10.1016/j.dib.2016.03.009

**Published:** 2016-03-10

**Authors:** Troy L. Campbell, Joe Quadrilatero

**Affiliations:** Department of Kinesiology, University of Waterloo, Waterloo, Ontario, Canada

**Keywords:** Skeletal muscle, Apoptosis, Autophagy, Doxorubicin

## Abstract

Skeletal muscle apoptosis and autophagy are catabolic processes that contribute to muscle atrophy during aging, disease, and following muscle injury. In this article, we present data on skeletal muscle apoptosis, autophagy, and morphology in C57BL/6 mice following doxorubicin administration. More specifically, time-course data on caspase-3, caspase-8, caspase-9, calpain, and cathepsin activity are presented, along with data on ATG7, p62, LC3-I, and LC3-II protein expression. Data on skeletal muscle reactive oxygen species (ROS) production, muscle morphology, as well as body and muscle weights are also presented.

**Specification Table**

**Table:** 

Subject area	*Biology*
More specific subject area	*Skeletal muscle, apoptosis, autophagy*
Type of data	*Images, graphs, figures*
How data was acquired	*Microscopy, spectrofluorometry, immunoblotting*
Data format	*Analyzed*
Experimental factors	*C57BL/6 mice received a single intraperitoneal injection of doxorubicin and were sacrificed at given time points.*
Experimental features	*C57BL/6 mice were sacrificed prior to (i.e. day 0) and 1, 3, 5, 7, and 9 days after a single intraperitoneal injection of doxorubicin. Different skeletal muscles were isolated and weighed, and samples prepared for microscopy, spectrofluorometric assays, and immunoblotting.*
Data source location	*University of Waterloo, Waterloo, Ontario, Canada*
Data accessibility	*All data are provided with this article*

**Value of the data**

● Provides a simultaneous assessment of skeletal muscle apoptosis, autophagy, and morphology in response to systemic doxorubicin administration.
● Provides time-course data on skeletal muscle morphology and degradative processes following doxorubicin administration.
● Valuable for researchers interested in the relationship between apoptosis and autophagy in skeletal muscle.
● Help other researchers determine the utility of doxorubicin as a myotoxic agent.

## 1 Data

Here we present data regarding the ability of systemic doxorubicin administration to induce skeletal muscle atrophy and morphological changes. Given that apoptosis and autophagy can impact muscle morphology and wasting [1], [2] we also provide data on apoptotic and autophagic signaling in several muscles following doxorubicin administration.

### 1.1 Body and muscle weights following doxorubicin administration

**Fig. 1:**
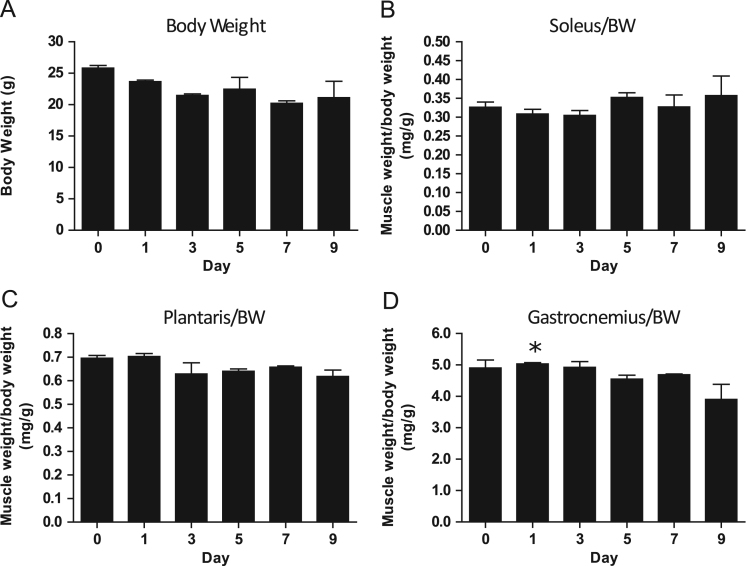
Body weight (A), as well as soleus (B), plantaris (C), and gastrocnemius (D) muscle weights normalized to body weight (BW) prior to (day 0) and following (day 1, 3, 5, 7, 9) doxorubicin administration (*n*=3). *p<0.05, significant difference compared to day 9.

There was a trend (*p*=0.07) towards a significant decrease in body weight after doxorubicin administration (Fig. 1A). There were no statistically significant differences in soleus, plantaris, or gastrocnemius weights with doxorubicin. Further, there were no significant differences in muscle weights when normalized to body weight for both the soleus (Fig. 1B) or plantaris (Fig. 1C) following doxorubicin administration. However, post hoc analysis revealed a significant (*p*<0.05) difference in gastrocnemius weight when normalized to body weight at day 1 compared to day 9 (Fig. 1D).

### 1.2 Apoptotic enzyme activity and reactive oxygen species (ROS) generation following doxorubicin treatment

**Fig. 2:**
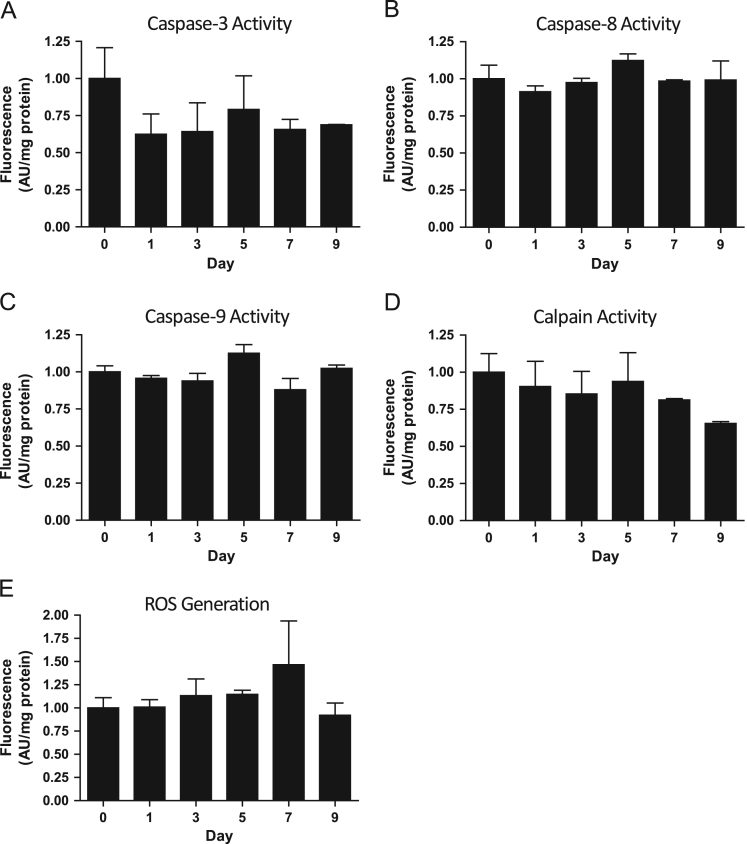
Caspase-3 (A), caspase-8 (B), caspase-9 (C), and calpain (D) activity, as well as ROS generation (E) data from mixed gastrocnemius prior to (day 0) and following (day 1, 3, 5, 7, 9) doxorubicin administration (*n*=3).

There were no statistically significant differences in caspase-3 (Fig. 2A), caspase-8 (Fig. 2B), caspase-9 (Fig. 2C), or calpain (Fig. 2D) activity, nor ROS production (Fig. 2E) in mixed gastrocnemius between any of the measured time points (Day 0, 1, 3, 5, 7, and 9) following doxorubicin administration.

### 1.3 Autophagic protein expression and cathepsin activity after doxorubicin administration

**Fig. 3:**
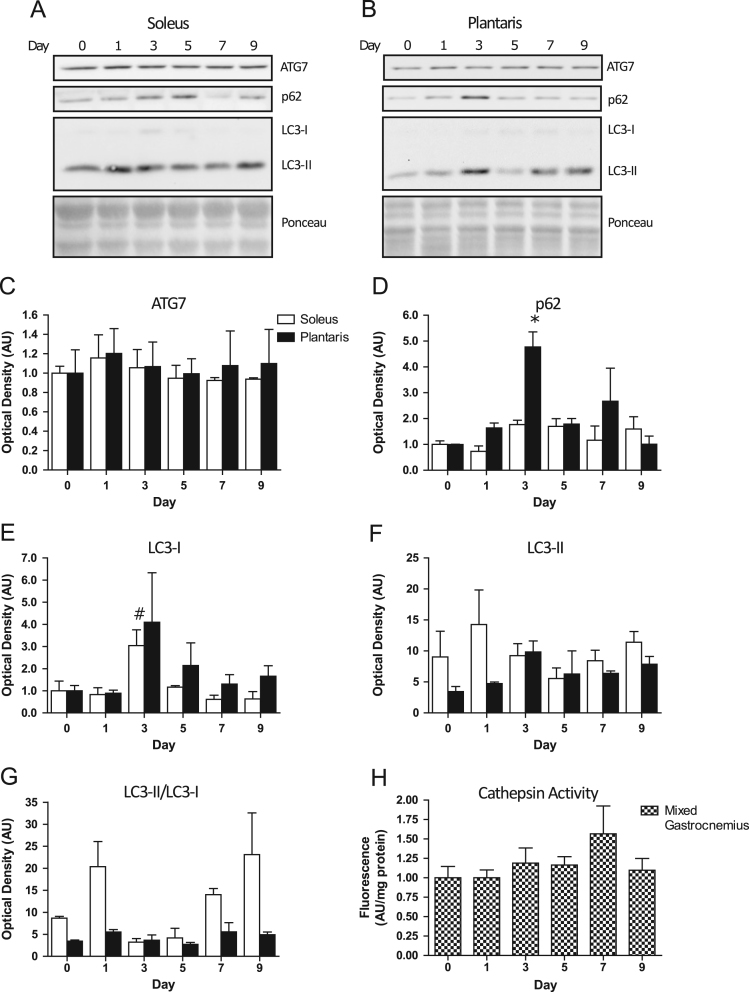
Representative immunoblots from soleus (A) and plantaris (B) of ATG7, p62, LC3-I, and LC3-II protein expression prior to (day 0) and following (day 1, 3, 5, 7, 9) doxorubicin administration. Quantitative analysis of ATG7 (C), p62 (D), LC3-I (E), and LC3-II (F) protein, as well as the LC3-II/LC3-I protein ratio (G) in soleus and plantaris before and after doxorubicin treatment (*n*=3). Quantitative analysis of cathepsin activity (H) from mixed gastrocnemius prior to and following doxorubicin injection (*n*=3). *p<0.05, significant difference compared to day 0, 1, 5, and 9. ^#^*p*<0.05, significant difference compared to day 1.

There were no differences in ATG7 (Fig. 3A and C) or p62 (Fig. 3A and D) protein between any of the measured time points in the soleus following doxorubicin administration. Post hoc analysis revealed a significant (*p*<0.05) increase in LC3-I protein in soleus on day 3 post-doxorubicin injection compared to day 1 (Fig. 3A and E). There was no statistically significant difference in LC3-II protein (Fig. 3A and F), nor the LC3-II/LC3-I protein ratio (Fig. 3A and G) in the soleus at any time point following doxorubicin administration. There were no significant differences in ATG7 protein (Fig. 3B and C) at any time point after doxorubicin administration in the plantaris muscle. However, p62 protein (Fig. 3B and D) was significantly (*p*<0.05) higher on day 3 compared to day 0, 1, 5, and 9. There were no significant differences in LC3-I protein (Fig. 3B and E), LC3-II protein (Fig. 3B and F), or the LC3-II/LC3-I protein ratio (Fig. 3B and G) in the plantaris at any time point following doxorubicin administration. Finally, there were no statistically significant differences in cathepsin activity between time points in mixed gastrocnemius following doxorubicin administration (Fig. 3H).

### 1.4 Qualitative analysis of muscle morphology after doxorubicin exposure

**Fig. 4:**
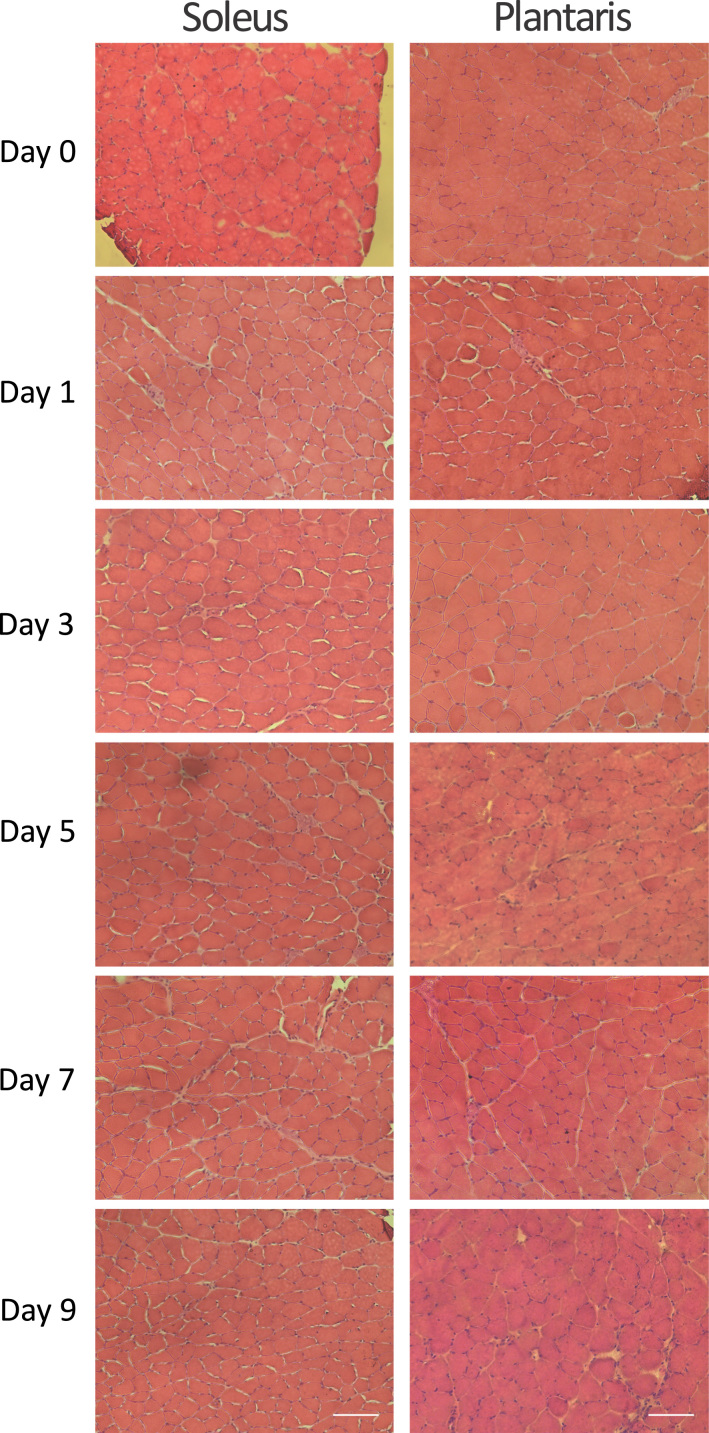
Representative H & E stained muscle cross-sections from soleus and plantaris muscle prior to (day 0) and following (day 1, 3, 5, 7, 9) doxorubicin administration. Scale bar=100 µm.

Visual inspection of soleus and plantaris cross-sections stained with hematoxylin & eosin (H & E) showed no obvious signs of muscle damage or changes in muscle morphology at any time point after doxorubicin administration (Fig. 4). Additionally, there were no apparent increases in the presence of centralized nuclei after doxorubicin administration.

## 2 Experimental design, materials and methods

### 2.1 Experimental design

Male C57BL/6 mice between the ages of 13–17 weeks were sacrificed by cervical dislocation prior to (i.e. day 0) and 1, 3, 5, 7, or 9 days (*n*=3 for each time point) following a single intraperitoneal injection of doxorubicin at a concentration of 20 mg/kg body weight. This dose has previously been shown to induce atrophy, damage, oxidative stress, and apoptotic and autophagic signaling in skeletal muscle at 24 h or 48 h following administration [3], [4], [5], [6].

### 2.2 Muscle extraction and sample preparation

After sacrifice, the soleus, plantaris, and gastrocnemius (mixed) were extracted from both hindlimbs. A sample of the entire circumference from the middle portion of one soleus and one plantaris was mounted in Optimal Cutting Temperature (OCT) compound and frozen in liquid nitrogen-cooled isopentane. The remaining soleus and plantaris, as well as the mixed gastrocnemius, were individually frozen in liquid nitrogen and stored at −80 °C.

Soleus and plantaris muscle samples used for immunoblotting were homogenized in ice-cold lysis buffer (20 mM HEPES, 10 mM NaCl, 1.5 mM MgCl_2_, 1 mM DTT, 20% glycerol and 0.1% Triton X-100; pH 7.4) containing protease inhibitors (Complete Cocktail; Roche Diagnostics). Mixed gastrocnemius muscle samples used for caspase, calpain, and cathepsin activity assays were homogenized in lysis buffer but without protease inhibitors. Homogenates were then centrifuged for 10 min at 1000*g* at 4 °C and supernatants collected and stored at −80 °C. Mixed gastrocnemius samples for ROS assays were homogenized in ice-cold buffer (250 mM sucrose, 20 mM HEPES, 10 mM KCl, 1 mM EDTA, 1 mM EGTA, 1 mM DTT; pH 7.4) containing protease inhibitors (Complete Cocktail; Roche Diagnostics). Total protein concentrations were determined by the BCA protein assay.

### 2.3 Proteolytic enzyme activity

Caspase-3, -8, and -9 activity in mixed gastrocnemius homogenates were assessed using the substrates Ac-DEVD-AMC (Enzo Life Sciences), Ac-IETD-AMC (Sigma-Aldrich), and Ac-LEHD-AMC (Enzo Life Sciences), respectively, as previously performed [7]. Calpain activity was measured in mixed gastrocnemius homogenates using Suc-LLVY-AMC (Enzo Life Sciences) either with or without the calpain inhibitor Z-LL-CHO (Enzo Life Sciences). Calpain activity was calculated as the difference between the average fluorescence of wells with and without the inhibitor [7]. Cathepsin activity was determined in mixed gastrocnemius homogenates using z-FR-AFC (Enzo Life Sciences) as previously described [8]. Fluorescence for all enzymes was determined using a SPECTRAmax Gemini XS microplate spectrofluorometer (Molecular Devices), then normalized to total protein content, and expressed as arbitrary units per milligram of protein.

### 2.4 Reactive oxygen species generation

Reactive oxygen species (ROS) generation was assessed in mixed gastrocnemius using dichlorofluorescein-diacetate (DCFH-DA) (Life Technologies) [9]. Fluorescence was determined using a SPECTRAmax Gemini XS microplate spectrofluorometer (Molecular Devices). Fluorescence was normalized to total protein content and presented as arbitrary units per milligram of protein.

### 2.5 Immunoblotting analyses

Immunoblotting was performed using soleus and plantaris muscle samples as previously described [10]. Briefly, equal amounts of protein were loaded into 12% SDS-PAGE gels, separated by electrophoresis, and transferred onto PVDF membranes (BioRad). Blocked membranes were incubated in primary antibody against ATG7 (Cell Signaling Technology), LC3B (Cell Signaling Technology), and p62 (Progen Biotechnik) overnight at 4 °C, washed, and incubated with the appropriate horseradish peroxidase-conjugated secondary antibody (Santa Cruz Biotechnology) for 1 h at room temperature. Protein bands were imaged using Clarity Western ECL Substrate (BioRad) and the ChemiGenius 2 Bio-Imaging System (Syngene). Membranes were also stained with Ponceau S (Sigma-Aldrich) to verify equal protein loading and quality of protein transfer.

### 2.6 Histochemical analyses

Soleus and plantaris muscle cross-sections mounted on microscope slides were first stained with hematoxylin for 30 s, after which excess hematoxylin was quickly removed, and slides washed in distilled water. Cross-sections were then stained with eosin for 90 s, after which excess eosin was quickly removed. Slides were then treated with graded ethanol incubations, a xylene clearing step, and finally coverslips mounted with permount.

### 2.7 Statistical analyses

All quantitative data was analyzed using 1-way ANOVAs and Tukey׳s post hoc tests, with *p*<0.05 being considered statistically significant.

## References

[1] Fanzani A., Conraads V.M., Penna F., Martinet W. (2012). Molecular and cellular mechanisms of skeletal muscle atrophy: an update. J. Cachexia Sarcopenia Muscle.

[2] Sandri M. (2010). Autophagy in skeletal muscle. FEBS Lett..

[3] Smuder A.J., Kavazis A.N., Min K., Powers S.K. (2011). Exercise protects against doxorubicin-induced markers of autophagy signaling in skeletal muscle. J. Appl. Physiol..

[4] Smuder A.J., Kavazis A.N., Min K., Powers S.K. (2011). Exercise protects against doxorubicin-induced oxidative stress and proteolysis in skeletal muscle. J. Appl. Physiol..

[5] Min K., Kwon O.S., Smuder A.J., Wiggs M.P., Sollanek K.J., Christou D.D., Yoo J.K., Hwang M.H., Szeto H.H., Kavazis A.N., Powers S.K. (2015). Increased mitochondrial emission of reactive oxygen species and calpain activation are required for doxorubicin-induced cardiac and skeletal muscle myopathy. J. Physiol..

[6] Kavazis A.N., Smuder A.J., Powers S.K. (2014). Effects of short-term endurance exercise training on acute doxorubicin-induced FoxO transcription in cardiac and skeletal muscle. J. Appl. Physiol..

[7] Mitchell A.S., Smith I.C., Gamu D., Donath S., Tupling A.R., Quadrilatero J. (2015). Functional, morphological, and apoptotic alterations in skeletal muscle of ARC deficient mice. Apoptosis.

[8] Bloemberg D., McDonald E., Dulay D., Quadrilatero J. (2014). Autophagy is altered in skeletal and cardiac muscle of spontaneously hypertensive rats. Acta Physiol. (Oxford).

[9] McMillan E.M., Quadrilatero J. (2011). Differential apoptosis-related protein expression, mitochondrial properties, proteolytic enzyme activity, and DNA fragmentation between skeletal muscles. Am. J. Physiol. Regul. Integr. Comp. Physiol..

[10] Campbell T.L., Mitchell A.S., McMillan E.M., Bloemberg D., Pavlov D., Messa I., Mielke J.G., Quadrilatero J. (2015). High-fat feeding does not induce an autophagic or apoptotic phenotype in female rat skeletal muscle. Exp. Biol. Med. (Maywood).

